# Supplementary material for: An estimate of the area of occupancy and population size of Brachycephalus tridactylus (Anura: Brachycephalidae) to reassess its conservation status, with a proposal for conservation measures
Source: PeerJ. 2021 Dec 22;9:e12687. doi: 10.7717/peerj.12687 (PMC8710056; doi:10.7717/peerj.12687)
Supplement: Supplemental Information 1 [file peerj-09-12687-s001.docx]

Table S1. Localities without records of *Brachycephalus tridactylus* searched from 2016–2020.

| Locality | Geographic coordinates (DATUM WGS84) | Altitude (m) above sea level | | Number of visits |
| --- | --- | --- | --- | --- |
|  |  | Searched for the species | With forests |  |
| Barra da Cruz, municipality of Bocaiúva do Sul, Paraná | 25°03’13”S, 48°50’22”W | 875–885 | 875–885 | 1 |
| Capivari Grande, Serra do Capivari, boundary of the municipalities of Campina Grande do Sul and Antonina, Paraná | 25°08’20”S, 48°49’19”W | 1,560–1,630 | 1,560–1,630 | 1 |
| Access to Capivari Grande, Serra do Capivari, boundary of the municipalities of Campina Grande do Sul and Antonina, Paraná | 25°08’00”S, 48°49’14”W | 1,270–1,560 | 1,525–1,560 | 2* |
| Capivari Mirim, Serra do Capivari, boundary of the municipalities of Campina Grande do Sul and Antonina, Paraná | 25°09’02”S, 48°49’41”W | 1,550–1,575 | 1,550–1,575 | 1 |
| Access to Capivari Mirim, Serra do Capivari, boundary of the municipalities of Campina Grande do Sul and Antonina, Paraná | 25°09’01”S, 48°50’20”W | 910–1,550 | 1,500–1,550 | 1** |
| Córrego Ribeiro, municipality of Barra do Turvo, São Paulo | 24°42’20”S, 48°31’16”W | 580–620 | 615–620 | 1 |
| Entroncamento Teba, municipality of Campina Grande do Sul, Paraná | 25°01’29”S, 48°37’10”W | 785–800 | 785–800 | 2 |
| Near the Entroncamento Teba, municipality of Campina Grande do Sul, Paraná | 25°01’47”S, 48°36’52”W | 710–740 | 710–740 | 1 |
| PCH Novo Horizonte, left bank of Rio Capivari Cachoeira, municipality of Bocaiúva do Sul, Paraná | 24°59’22”S, 48°36’28”W | 660–680 | 660–680 | 1 |
| PCH Novo Horizonte, right bank of Rio Capivari Cachoeira, municipality of Campina Grande do Sul, Paraná | 25°00’41”S, 48°37’47”W | 670–710 | 670–710 | 1 |
| Ribeirão Grande, municipality of Campina Grande do Sul, Paraná | 25°06’10”S, 48°47’14”W | 740 | 740 | 1 |
| Serra do Itapitangui, on the border of municipalities of Cananéia and Jacupiranga, São Paulo | 24°57’36”S, 48°01’45”W | 190–940 | 255–940 | 3 |
| Serra do Saltinho, municipality of Iporanga, São Paulo | 24°41’35”S, 48°32’05”W | 655–810 | 655–810 | 1 |
| Serra Gigante, municipality of Guaraqueçaba, Paraná | 25°09’16”S, 48°10’33”W | 30–810 | 30–810 | 1 |
| Torre Cajati, on the border of the municipalities of Jacupiranga and Cajati, São Paulo | 24°47’26”S, 48°07’09”W | 740–840 | 775–840 | 3 |

* We made eight additional field trips before 2016.

** We made one additional field trip before 2016.
